# Non-*Lactobacillus*-Dominated Vaginal Microbiota Is Associated With a Tubal Pregnancy in Symptomatic Chinese Women in the Early Stage of Pregnancy: A Nested Case–Control Study

**DOI:** 10.3389/fcimb.2021.659505

**Published:** 2021-07-07

**Authors:** Xiao-Feng Ruan, Ying-Xuan Zhang, Si Chen, Xiao-Rong Liu, Fang-Fang Zhu, Yan-Xi Huang, Xiao-Jing Liu, Song-Ping Luo, Gao-Pi Deng, Jie Gao

**Affiliations:** ^1^ First Clinical Medical College, Guangzhou University of Chinese Medicine, Guangzhou, China; ^2^ Department of Gynecology, Second Affiliated Hospital of Guangzhou University of Chinese Medicine, Guangzhou, China; ^3^ Department of Gynecology, First Affiliated Hospital of Guangzhou University of Chinese Medicine, Guangzhou, China

**Keywords:** symptomatic early pregnancy, tubal pregnancy, vaginal microbiota, *Lactobacillus*, non-*Lactobacillus* dominated microbiota

## Abstract

The features of the vaginal microbiota (VM) community can reflect health status, and they could become new biomarkers for disease diagnosis. During pregnancy, domination of bacteria of the genus *Lactobacillus* in the VM community is regarded as a keystone because they stabilize the VM by producing antimicrobial compounds and competing adhesion. An altered VM composition provides a marker for adverse pregnancy outcomes. This nested case–control study aimed to characterize the VM in women with a tubal pregnancy (TP) presenting with pain and/or uterine bleeding in early pregnancy. Chinese women with a symptomatic early pregnancy of unknown location were the study cohort. 16S rDNA gene-sequencing of V3–V4 variable regions was done to assess the diversity, structures, taxonomic biomarkers, and classification of the VM community. The primary outcome was the location of the early pregnancy. The VM community in women with a TP showed higher diversity (PD-whole-tree, median: 8.26 *vs*. 7.08, *P* = 0.047; Shannon Diversity Index, median: 1.43 *vs* 0.99, *P* = 0.03) and showed different structures to those in women with an intrauterine pregnancy (IUP) (*R* = 0.23, *P* < 0.01). Bacteria of the genus *Lactobacillus* were significantly enriched in the IUP group, whereas bacteria of the genera *Gardnerella* and *Prevotella* were significantly enriched in the TP group. *Lactobacillus* abundance could be used to classify the pregnancy location (AUC = 0.81). Non-*Lactobacillus*-dominated microbiota (≤ 0.85% *Lactobacillus*) was significantly associated with a TP (adjusted odds ratio: 4.42, 95% confidence interval: 1.33 to 14.71, *P* = 0.02). In conclusion, among women with a symptomatic early pregnancy, a higher diversity and lower abundance of *Lactobacillus* in the VM is associated with a TP.

## Introduction

An ectopic pregnancy occurs when a fertilized egg implants and grows outside the main cavity of the uterus. An ectopic pregnancy most often occurs within a fallopian tube. This type of ectopic pregnancy is called a “tubal pregnancy” (TP). TP is the leading cause of hemorrhage-related mortality, accounting for 2.7% of pregnancy-related mortality (Creanga et al., 2017).

Early screening of high-risk patients, as well as the early diagnosis and management of an unruptured TP, can reduce morbidity and mortality and preserve fertility. Although half of TP patients have no definite risk factor, several risk factors have been identified (Silva et al., 2006; Barnhart et al., 2011; ACOG, 2018). Pelvic inflammatory disease (PID) is one of the most important risk factors. Retrospective case–control studies have shown that the prevalence of fallopian tube damage increases after continuous exposure to PID (13% after one exposure, 35% after two exposures, and 75% after three exposures), which may lead to an increase in the incidence of TP (Bjartling et al., 2007; Bakken, 2008). However, assessment of the effect of a genital infection upon reproductive outcome is difficult due to the lack of reliable methods for measuring genital infections (Onan et al., 2005; AOCG, 2018; Elson et al., 2016). Conversely, one nested case–control study with a sample size of 2026 found that pain as the presenting symptom (odds ratio (OR): 1.16, 95% confidence interval (CI): 0.92–1.48) and bleeding as the presenting symptom (OR: 1.34, 95%CI:1.04–1.78) were risk factors for an ectopic pregnancy (Barnhart et al., 2006). More attention should be paid to such symptomatic women in the early stages of pregnancy. Therefore, finding inflammation-related biomarkers associated with TP risk in symptomatic women in early pregnancy is important.

DNA-sequencing (DNA-seq) methods have enabled culture-independent analyses of complex microbial communities. In this way, they have provided an integrative view and a much broader picture of the vaginal microbiota (VM). Ranging from nutrient acquisition, metabolic activity, to immune homeostasis, the VM, along with host cells, constitutes complex and interactive processes. The microbiota can affect female physiology, and female physiology can affect the composition and function of the VM (Smith and Ravel, 2017). The features of the VM community can reflect the health of women, and the VM could become a new biomarker for disease diagnosis.

In women with a normal pregnancy, the VM community shifts to become more dominated by *Lactobacillus*, less diverse, and highly stable, and this transition occurs in early pregnancy (Ravel et al., 2011; Hickey et al., 2012; DiGiulio et al., 2015; Freitas et al., 2017; Smith and Ravel, 2017; Blostein et al., 2020; Tsonis et al., 2020). During pregnancy, this dominance by *Lactobacillus* is regarded as a keystone in the VM community because it stabilizes the VM by producing antimicrobial compounds (e.g., lactic acid, hydrogen peroxide, and bacteriocins) and competing adhesion (Borges et al., 2014; Greenbaum et al., 2019). An altered VM composition provides a marker for adverse pregnancy outcomes. In early pregnancy, a decreased number of *Lactobacillus* alone with increased abundance of anaerobes and a highly diverse VM community is associated with miscarriage and preterm birth (Romero et al., 2014; DiGiulio et al., 2015; Callahan et al., 2017; Haque et al., 2017; Freitas et al., 2018; Fettweis et al., 2019; Al-Memar et al., 2020). Among women undergoing *in vitro* fertilization (IVF) treatment, infertility based on fallopian tube factors is more prevalent in those with an abnormal VM, which is characterized by a shift from a *Lactobacillus* -dominated state to a state of increased heterogeneous anaerobes (Haahr et al., 2016; Haahr et al., 2019). However, the association between the VM in early pregnancy and a TP is not known.

We wished to analyze whether particular characteristics of a VM community in early pregnancy were linked to a TP. We undertook a study using 16S rRNA gene sequencing (16S rRNA gene-seq) among women in early pregnancy who were initially considered to have a pregnancy of unknown location (PUL), and subsequently diagnosed with an intrauterine pregnancy (IUP) or TP. Taking into account the differences in vaginal microbiota between ethnicity and that this study only included Chinese women, the aim of this study is to evaluate the potential association between vaginal microbiota and pregnancy location.

## Materials and Methods

### Ethical Approval of the Study Protocol

The study protocol was approved (ZYYECK2017-060; approval date: 6 February 2018) by the Ethics Committee of the First Affiliated Hospital of Guangzhou University of Chinese Medicine (Guangzhou, China). All participants provided written informed consent.

### Inclusion Criteria

The inclusion criteria were pregnant women: (i) aged ≥18 years; (ii) with PUL [positive pregnancy test but no evidence on transvaginal ultrasound of an IUP or TP (Barnhart et al., 2011)]; (iii) who presented with pain and/or bleeding; and (iv) with a gestational age (from the last day of the last menstruation) of 4–8 weeks.

### Exclusion Criteria

The exclusion criteria were pregnant women: (i) who had taken antibiotics within 30 days before sample collection; (ii) had sexual activity, vaginal douching, or recorded use of vaginal medications within 48 h before sample collection; or (iii) with vulvovaginal candidiasis, acute inflammation, or cancer.

### Study Design

This nested case–control study was based on follow-up of a symptomatic cohort of Chinese women in early pregnancy. During May to December 2018, we enrolled women who were initially considered to have PUL at the First Affiliated Hospital of Guangzhou University of Chinese Medicine.

Women were followed up until the definitive location of the pregnancy could be made. “Cases” were defined as women who were diagnosed definitively with a TP. “Controls” were defined as women who were diagnosed definitively with an IUP in the same cohort. The diagnosis of a TP and IUP were based on medical history, clinical symptoms, physical examination, serial serum levels of human chorionic gonadotropin, and the findings of transvaginal ultrasound. An IUP was validated by a yolk sac or embryo within an intrauterine gestational sac (Barnhart et al., 2011). A TP was confirmed by laparoscopy and histopathology. Controls matched with cases for presenting with age (± 5 years) and gestational age of sample collection (± 7 days) at a ratio of 2:1 (Filion et al., 2016).

### Collection of Clinical Data and Samples

At their initial visit, participants completed a questionnaire survey (including details on sociodemographic characteristics, past medical/reproductive history, and lifestyle), measurement of height and weight (without shoes or clothes), speculum examination, and collection of vaginal secretions. Pelvic inflammatory disease was defined as all outpatient, inpatient, and emergency treatment (Brunham et al., 2015). Collection of vaginal secretions from each participant was undertaken by a very experienced obstetrician. A sterile speculum without lubrication was inserted into the vaginal canal. Three sterile swabs (Improve Medical, Guangzhou, China) with triplicates were applied five times to both sides of the mid-vaginal canal. Swabs were placed in a sterile tube separately. One swab was used for screening and excluding vulvovaginal candidiasis by wet mount microscopy (Workowski and Bolan, 2015). One swab was sent immediately to measure the pH of the vaginal secretion. One swab was frozen at −20°C within 4 h after collection, transported to a laboratory, and then stored at −80°C until DNA extraction.

### pH

Vaginal pH was measured using pH test paper (Sanaisi Scientific Instruments, Jiangsu, China) on an automatic vaginitis detection system (bPR-2014A; Bioperfectus Technologies, Jiangsu, China) ranging from 3.8 to 5.4 (incremental change was 0.2).

### Extraction, Sequencing, and Data Processing of 16S rDNA

The main processes for extraction and sequencing of DNA were done in eight steps. First, genomic DNA was extracted using the spin-column method with the Hipure Bacterial DNA kit (Magen, Guangzhou, China). Second, quality control was undertaken using Qubit 2.0 (Agilent Technologies, Santa Clara, CA, USA) with a dsDNA HS Assay kit (Life Technologies) and agarose gel electrophoresis. Third, samples which passed the quality-control test were employed for library construction. Universal primers for the variable regions of 16S V3–V4 were amplified and purified by a multiplex polymerase chain reaction assay (NEBnext^®^ Ultra™ II Q5^®^ Master Mix, New England Biolabs, Ipswich, MA, USA). The quality of the library was checked by Qubit 2.0 and the 2200 Tapestation system (Agilent Technologies). Fourth, samples were barcoded and mixed before pooling. Fifth, paired-end sequencing was done on the MiSeq^®^ Sequencing System (Illlumina, San Diego, CA, USA) with MiSeq Reagent Kit v3. Sixth, the raw sequencing data (raw reads) were denoised and filtered. Seventh, forward and reverse clean reads were coalesced into tags (paired-end reads) using fast length adjustment of short reads (FLASH) (Johns Hopkins University, Baltimore, MD, USA) (Magoc and Salzberg, 2011). Eighth, chimeras were removed using ultra-fast sequence analysis (USEARCH 61) (drive5; BioInformatics, Arlington, VA, USA). Finally, reads were filtered out if their length was <200 bp.

We pre-clustered the remaining high-quality tags into operational taxonomic units (OTUs) using a 2% single-linkage pre-clustering methodology to remove spurious OTUs (Huse et al., 2010). Next, we used the UCLUST algorithm to cluster the remaining tags into OTUs based on 97% nucleotide similarity (Edgar, 2010). SILVA (SILVA_132_QIIME_release; (Quantitative Insights Into Microbial Ecology (QIIME); http://qiime.org/index.html/) was used to classify the seed sequences of each OTU into specific taxa, even at the species-level classification of *Lactobacillus*.

### Data Analyses

We used alpha diversity (phylogenetic diversity (PD)-whole-trees and Shannon Diversity Index) to analyze the within-community diversity. Beta diversity (weighted UniFrac distances) was employed to analyze the variation in community composition (Lozupone et al., 2011). Alpha diversity and beta diversity were calculated using QIIME (Caporaso et al., 2010). Principal component analysis (PCoA) was applied to further discover the community structure using statistical analysis of metagenomic profiles (STAMP). Analysis of similarities (ANOSIM) with 999 permutations was done using PRIMER-e 7.0 (www.primer-e.com/) to test the significant separation between groups in PCoA (Barrenha et al., 2017).

Linear discriminant analysis (LDA) effect size (LEfSe) methods with default parameters (alpha value for Wilcoxon tests was 0.05, the threshold on the logarithmic LDA score was 4.0) were implemented to further compare and visualize the significant differences in taxa (Segata et al., 2011).

In reference to the work of Moreno and colleagues (Moreno et al., 2016), to construct the classification of the VM in TP, we undertook five trials of 10-fold cross-validated classification and regression tree (CART) using the relative abundance of taxa in the Python Sklearn module (sklearn 0.21.3; http://scikit-learn.org/). The average area under the receiver operating characteristic curve (AUC) for five trials were used to compare the accuracy of various classifiers.

Multivariate logistic regression analysis was done to evaluate the associations between the VM and TP with EmpowerStats (www.empowerstats.com/). Non-adjusted and multivariate adjusted models were listed. Covariances were adjusted so that, upon addition to this model, the matched odds ratio was changed by ≥10% (Kernan et al., 2000).

A heatmap of the composition of VM taxa was drawn using Pearson correlation distance and complete-linkage hierarchical clustering with the pheatmap package within R (v1.0.8; http://cran.r-project.org/web/packages/pheatmap/). To better visualize the diversity found in all participants (even those with a highly skewed taxa proportion) we used the log_10_-transformed relative abundance of each taxon.

Statistical analyses for the cohort characteristics and indices of alpha diversity were undertaken using SPSS 22.0 (IBM, Armonk, NY, USA). Differences between groups were calculated by the Student’s *t*-test for continuous variables with a normal distribution, Wilcoxon test for skewed continuous variables, and the chi-squared test or Fisher’s exact test for categorical variables. *P* < 0.05 (two-tailed) was considered significant.

## Results

### Characteristics of the Cohort and Samples

**Figure 1:**
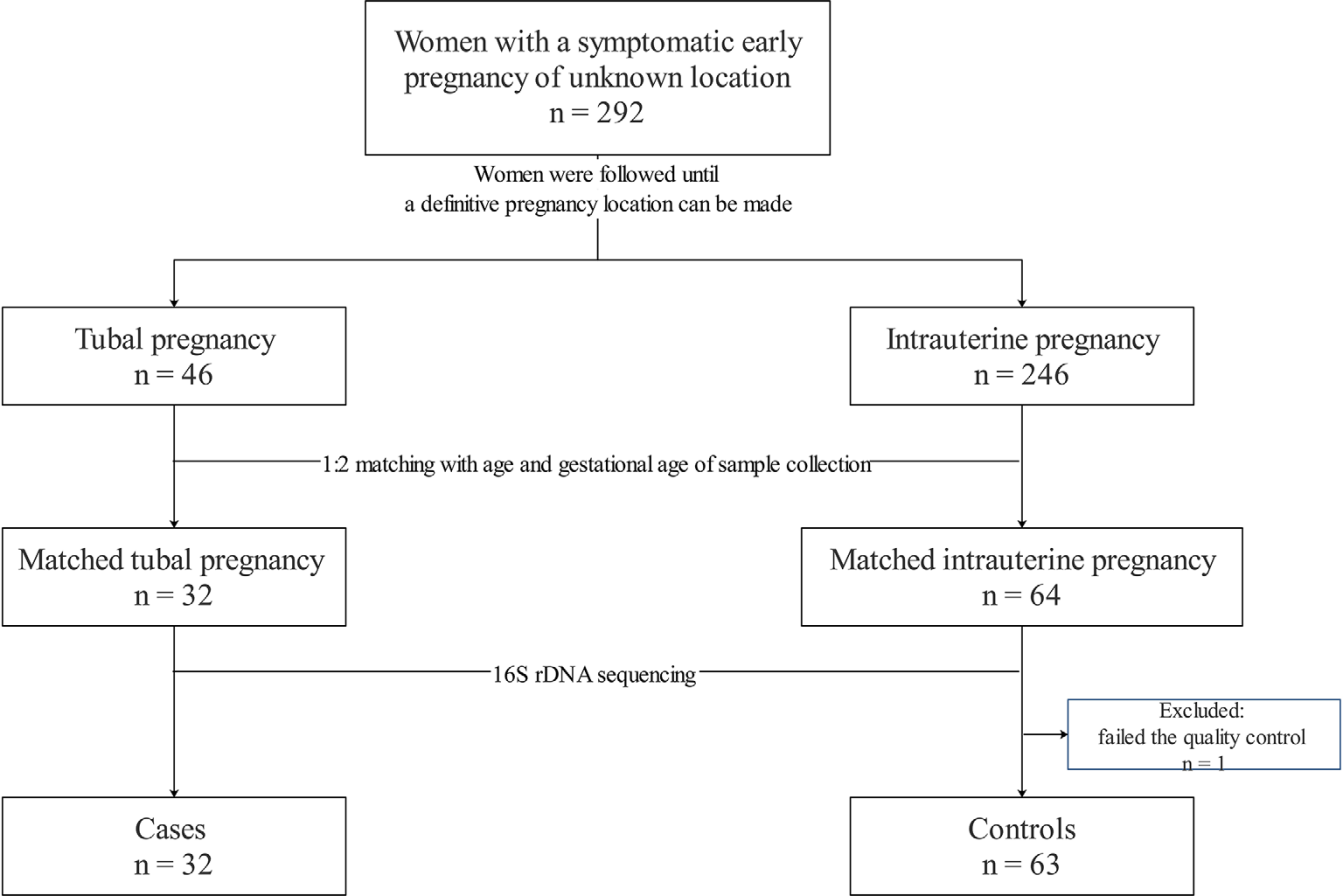
Flowchart of the study.

The cohort was comprised of 292 women with symptomatic early PUL, 46 of whom were diagnosed subsequently with a TP, and the remainder of whom diagnosed with an IUP. During the matching procedure, 32 women with a TP were 1:2 matched with 64 women with an IUP. However, one sample from a woman with an IUP failed the quality-control test and was excluded from the study. Ultimately, the final sample size for analysis was 32 cases and 63 controls (**Figure 1**).

The characteristics of the study cohort are described in **Table 1**. The mean age of the total study cohort was 29.6 ± 5.4 years. The gestational age of sample collection was 47.4 ± 7.6 days. Women with a TP were more likely to have uterine bleeding (81.2% *vs* 60.3%, *P* = 0.04), a shorter menstrual cycle (29.6 ± 3.8 *vs* 34.9 ± 10.2, *P* < 0.01), a previous ectopic pregnancy (18.8% *vs* 4.8%, *P* = 0.03), previous pelvic infection (53.1% *vs* 15.9%, *P* < 0.01), and higher vaginal pH (4.7 ± 0.4 *vs* 4.5 ± 0.3, *P* < 0.01) than those with an IUP. There were no significant differences in body mass index (BMI), gravidity, previous spontaneous abortion, previous surgery (uterine cavity, fallopian tube s, or pelvic), or tobacco-smoking status between the IUP group and TP group (P > 0.05).

**Table 1:** Characteristics of subjects with tubal pregnancy compared with those with intrauterine pregnancy.

	Tubal pregnancy	Intrauterine pregnancy	*P* value
Subjects	32	63	–
Gestational age, days	46.4 ± 6.9	48.0 ± 8.0	0.34
Age, years	30.1 ± 5.7	29.4 ± 5.4	0.56
BMI, kg/m^2^	22.0 ± 3.4	21.1 ± 2.8	0.15
Current smoker, yes	0	0	–
Uterine bleeding, yes	26 (81.2%)	38 (60.3%)	0.04*
Abdominal pain, yes	27 (84.4%)	42 (66.7%)	0.07
Menstrual cycle, days	29.6 ± 3.8	34.9 ± 10.2	<0.01*
Gravidity	3.0 (1.0-4.0)	2.0 (2.0-3.0)	0.41
Previous spontaneous abortion, yes	9 (28.1%)	29 (46.0%)	0.09
Previous ectopic pregnancy, yes	6 (18.8%)	3 (4.8%)	0.03^*^
Previous infertility, yes	6 (18.8%)	14 (22.2%)	0.70
Previous pelvic inflammatory disease, yes	17 (53.1%)	10 (15.9%)	<0.01*
Previous uterine cavity surgery, yes	18 (56.2%)	41 (65.1%)	0.40
Previous tubal surgery, yes	6 (18.8%)	4 (6.3%)	0.06
Previous pelvic surgery, yes	12 (37.5%)	21 (33.3%)	0.70
Vaginal environments			
Vaginal pH	4.7 ± 0.4	4.5 ± 0.3	<0.01*
non-*Lactobacillus* dominated microbiota (NLDM)	18 (56.2%)	8 (12.7%)	<0.01*

Continuous variables were presented as mean ± SD, categorical variables were expressed as percentages (%). P was calculated by t test for normally distributed continuous variables, chi-squared test, or Fisher’s exact test for categorical variables.

BMI body mass index.

*P < 0.05

### Women With a TP Showed a Higher Diversity in VM Composition Than That in Women With an IUP

After sequencing, denoising, and filtering, an average of 381,558 high-quality reads per sample was obtained. This value was deep enough for intensive analysis of taxa, especially for rare species (Kim et al., 2011; Ravel et al., 2011).

**Figure 2:**
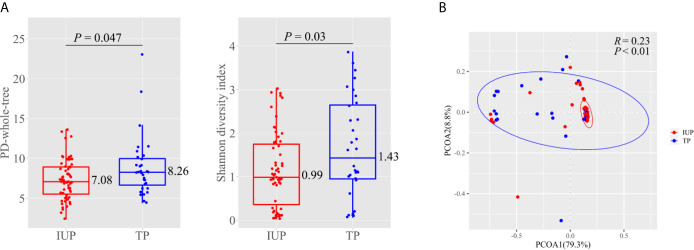
Diversity measures in subjects with intrauterine pregnancy and with tubal pregnancy. **(A)** Alpha diversity represented by PD-whole-tree and Shannon diversity index in subjects with intrauterine pregnancy and with tubal pregnancy. Boxes with inside line represented interquartile range (IQR) and median, whiskers represented values within 1.5 × IQR of the first and third quartiles, points represented individual subjects. *P* was calculated by Wilcoxon test. **(B)** Principal coordinate analysis (PCoA) based on weighted UniFrac distances between the subjects with intrauterine pregnancy and with tubal pregnancy. Points represented individual subjects, and ellipses represented 95% confidence intervals around the cluster centroid. ANOSIM calculated *R* and *P* to determine the significance of clustering. Red indicated intrauterine pregnancy and blue indicated tubal pregnancy.

At a sufficient sequencing depth, we compared alpha diversity (represented by PD-whole-trees and Shannon Diversity Index) in women with an IUP and women with a TP (**Figure 2A**). Compared with women with an IUP, women with a TP showed a higher PD-whole-tree value (median: 8.26 *vs* 7.08, *P* = 0.047) and Shannon Diversity Index (median: 1.43 *vs* 0.99, *P* = 0.03).

We undertook PCoA based on weighted UniFrac distances to further discover the diversity and community structure in women with an IUP or TP (**Figure 2B**). PCoA showed clustering, and the first principal coordinate (PC1) axes represented 79.3% of the total variations. ANOSIM showed that the dissimilarity between two groups was more significant than that within groups, which confirmed that the clustering was significant (*R* = 0.23, *P* < 0.01). These data indicated that, by considering the phylogeny as well as the abundance, women with a TP showed a different VM composition to that of women with an IUP.

**Figure 3:**
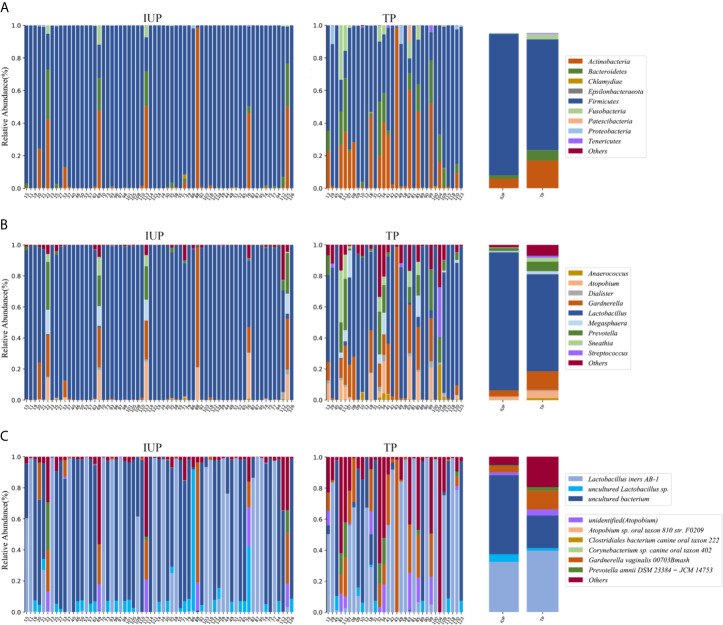
Vaginal microbial composition in subjects with intrauterine pregnancy and with tubal pregnancy. **(A)** Phylum level; **(B)** Genus level; **(C)** Species level composition in subjects with intrauterine pregnancy and with tubal pregnancy. Bar charts showing the vaginal microbial taxa composition in mean values (on the right side) and individual subjects with intrauterine pregnancy and with tubal pregnancy (on the left side).

The predominant taxa in the VM in women with an IUP were almost identical to those in women with a TP, but the relative abundance between the two groups was different (**Figure 3**). Firmicutes was the predominant phylum in women with an IUP (relative abundance: 91%) or a TP (relative abundance: 71%), but Actinobacteria was also common in TP (relative abundance: 18%). *Lactobacillus*, *Gardnerella*, and *Prevotella* were the predominant genera in both groups. In the IUP group, the relative abundance of the aforementioned genera was 89%, 4%, and 2%, respectively but, in the TP group, the relative abundance was 62%, 12%, and 6%. Bacteria of the genera *Atopobium* (4%), *Sneathia* (3%), and *Megasphaera* (2%) were also common in the TP group. Within the genus of *Lactobacillus*, *Lactobacillus iners AB-1*, *uncultured_bacterium*, and *uncultured Lactobacillus* were the major species in both groups.

### Non-*Lactobacillus* Dominated VM Was Associated With a TP

**Figure 4:**
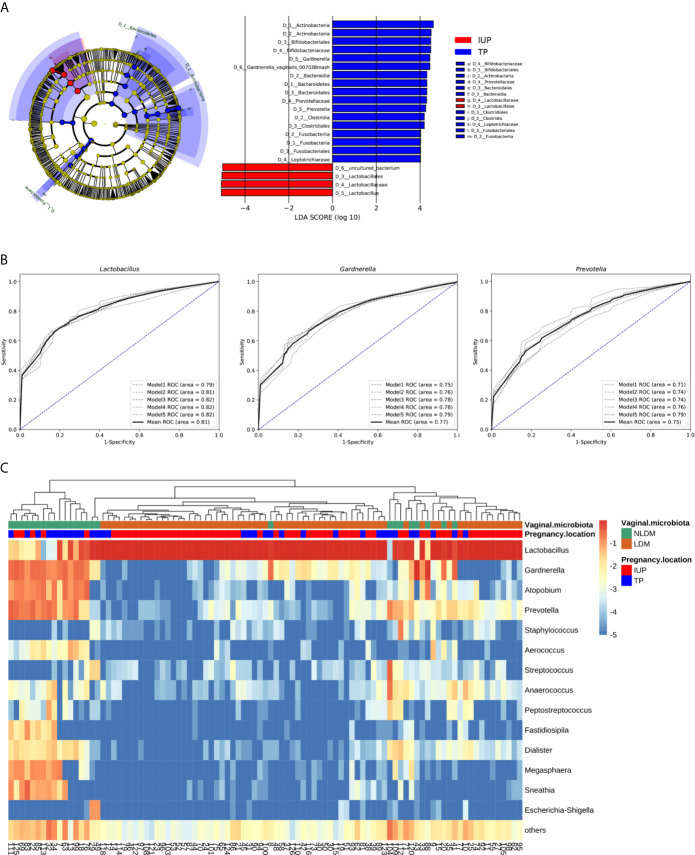
The establishment of *Lactobacillus* dominated microbiota (LDM) and non-*Lactobacillus* dominated microbiota (NLDM). **(A)** Cladogram and scores identified by linear discriminant analysis (LDA) using LEfSe. The nodes of cladogram from the inner to the outer circles indicated the abundant taxa from the kingdom (D0) to the species (D6) level. Colors represented the groups in which differentially abundant taxa were enriched (red indicated intrauterine pregnancy, blue indicated tubal pregnancy, yellow indicated non-significant), and the diameter of each node was proportional to the taxon’s abundance. The threshold on the logarithmic LDA score was 4.0. **(B)** Receiver operating characteristic curves for five trials of cross-validated CART of *Lactobacillu*, *Gardnerella*, and *Prevotella*. Black curve indicated the average ROC of the five trials (grey curves). The diagonal lines mark an area under the receiver operating characteristic curves of 0.5. **(C)** Heatmap of log10-transformed proportions of vaginal microbial taxa composition of 95 subjects clustered by pregnancy location and vaginal microbiota. The lower bar showed the pregnancy location of each subject (red indicated intrauterine pregnancy, blue indicated tubal pregnancy), while the upper bar showed vaginal microbiota (green indicated non-*Lactobacillus* dominated microbiota, orange indicated *Lactobacillus* dominated microbiota). The histogram showed the taxon log_10%_ relative abundance (darkest red indicated greatest abundance; light red and yellow indicated relatively less abundance; and green indicated low abundance or not present). Fourteen of the most abundant genera were showed.

We wished to further discover the potential taxonomic biomarkers which characterize the differences between an IUP and TP. We carried out LEfSe with a logarithmic LDA value of 4.0 (**Figure 4A**). We found genus *Lactobacillus*, order Lactobacillales, family Lactobacillaceae, and species *uncultured_bacterium* to be significantly enriched in the IUP group. Genus *Gardnerella*, phylum Actinobacteria, order Bifidobacteriales, and species *Gardnerella_vaginalis_00703Bmash* were significantly enriched in the TP group. Genus *Prevotella*, phylum Bacteroidetes, class Bacteroidia, order Bacteroidales, and family Prevotellaceae were also significantly enriched in the TP group. In addition, class Clostridia, order Clostridiale, and phylum Fusobacteria were significantly enriched in the TP group. Class Fusobacteriia, order Fusobacteriales, and family Leptotrichiaceae were also significantly increased in the TP group.

According to these analyses, we selected the relative abundance of three genera (*Lactobacillus*, *Gardnerella*, and *Prevotella*) to classify the pregnancy location (intrauterine pregnancy or tubal pregnancy). We carried out five trials of 10-fold cross-validated CART to classify samples. The average AUC for five trials of cross-validated CART of *Lactobacillus*, *Gardnerella*, and *Prevotella* was 0.81, 0.77, and 0.75, respectively (**Figure 4B**). The relative abundance of *Lactobacillus* was the most significant variable, and the best threshold was 85%. With regard to this model, the rule for classifying the pregnancy location was: if the relative abundance of *Lactobacillus* was > 85% the classification was an IUP; if the relative abundance of *Lactobacillus* was ≤ 85%, the classification was a TP. Based on these classifications, the VM of 95 women was divided into two groups: a relative abundance of *Lactobacillus* > 85% was termed “*Lactobacillus*-dominated microbiota” (LDM); a relative abundance of *Lactobacillus* ≤ 85% was classified as “non-*Lactobacillus*-dominated microbiota” (NLDM).

Compared with women with LDM, women with NLDM had a higher prevalence of a TP (56.2% *vs* 12.7%, *P* < 0.01). We used multivariate logistic regression analysis to evaluate the associations between NLDM and a TP. The association between NLDM and pregnancy location was robust in a crude model, minimally adjusted model, and fully adjusted model (**Table 2**). In the crude model, NLDM showed a positive association with an ectopic pregnancy [odds ratio (OR): 8.84, 95% confidence interval (CI): 3.19 to 24.48, *P* < 0.01]. In the minimally adjusted model (adjusted BMI), the result did not show an obvious change (OR: 9.46, 95%CI: 3.31 to 27.02, *P* < 0.01). Furthermore, in the fully adjusted model (adjusted for BMI, abdominal pain, vaginal pH, menstrual cycle, gravidity, previous spontaneous abortion, previous infertility, and previous uterine-cavity surgery), the correlation between NLDM and pregnancy location was stable (OR: 4.42, 95%CI: 1.33 to 14.71, *P* = 0.02).

**Table 2:** Multivariate logistic regression analysis for the relationship between non-Lactobacillus dominated microbiota and tubal pregnancy.

LDM	Crude Model	Minimally adjusted model	Fully adjusted model
	OR, 95% CI, *P*	OR, 95% CI, *P*	OR, 95% CI, *P*
	Ref	Ref	Ref
NLDM	8.84 (3.19, 24.48) <0.01	9.46 (3.31, 27.02) <0.01	4.42 (1.33, 14.71) 0.02

Crude Model adjusted for: None.

Minimally adjusted model adjusted for: BMI.

Fully adjusted model adjusted for: BMI, abdominal pain, vaginal pH, menstrual cycle, gravidity, previous spontaneous abortion, previous infertility, and previous uterine cavity surgery.

BMI, body mass index.

With respect to alpha diversity, consistent with the alpha diversity between the IUP group and TP group, women with NLDM showed higher PD-whole-tree (median: 8.26 *vs*. 7.03 P < 0.01) and Shannon Diversity Index (median: 2.66 *vs* 0.95, *P* < 0.01) than those in women with LDM. In terms of beta diversity, PCoA on weighted UniFrac distances showed clustering in PC1 (representing 79.3% of the total variation), and ANOSIM confirmed the clustering to be significant (*R* = 0.86, *P* < 0.01) (**Figure S1B**).

We created heatmaps to visualize the taxa composition of the VM of the total cohort clustered by pregnancy location (intrauterine pregnancy or tubal pregnancy) and VM (NLDM and LDM) (**Figure 4C**). Subjects with NLDM were almost associated with tubal pregnancy while LDM were associated with intrauterine pregnancy. Consistent with this analysis, the VM of women with NLDM was characterized by greater diversity and a relatively higher abundance of bacteria of genera *Gardnerella*, *Prevotella*, *Atopobium*, *Megasphaera*, and *Sneathia*. Women with LDM showed high levels of skew dominated by bacteria of the genus *Lactobacillus* and a small proportion of bacteria of other genera. These data supported the notion that higher evenness was the cause of greater diversity in a TP and in NLDM. Bacteria of the genus *Lactobacillus* in NLDM and LDM could be subdivided into *Lactobacillus iners AB-1*, *uncultured_bacterium*, *uncultured Lactobacillus*, *Lactobacillus crispatus*, *Lactobacillus gasseri*, and *Lactobacillus jensenii*. Most women had more than one species of *Lactobacillus* in their VM.

## Discussion

Before the pregnancy location could be visualized, the community structure of the VM in a TP was different to that of an IUP among women presenting with pain and/or uterine bleeding. A greater diversity and lower abundance of *Lactobacillus* in the VM was associated with a TP. As a potential valuable biomarker, the relative abundance of bacteria of the genus *Lactobacillus* could be used to classify the pregnancy location using a threshold of 85%. NLDM (≤85% *Lactobacillus*) was positively associated with a TP.

A TP is the main cause of hemorrhage-related mortality in early pregnancy (Creanga et al., 2017). Most studies have suggested that previous infection in the fallopian tube (which results in adhesions or cilia damage) leads to a TP months or years later. Women with a previous ectopic pregnancy, PID, tubal surgery, and infertility are associated with an increased risk of a TP. However, ≤50% of patients with a TP have no definite risk factor (Barnhart et al., 2006; ACOG, 2018).

Development of NGS technologies has enabled more in-depth understanding of the VM. The key role of the commensal microbiome in the lower genital tract upon maternal and neonatal health has been documented. An increasing number of studies have shown the VM composition to be closely related with reproductive outcomes. A healthy pregnancy is characterized by a *Lactobacillus* species-dominated, low-richness, and low-diversity VM composition (DiGiulio et al., 2015). Al Memar and colleagues (Al-Memar et al., 2020) found that a lower vaginal abundance of *Lactobacillus*, high diversity, and high richness of bacteria was positively related with first-trimester miscarriage. This phenomenon was observable if the pregnancy seemed viable on ultrasound before miscarriage. Brown and coworkers (Brown et al., 2018) reported that VM disorders characterized as depletion of *Lactobacillus* and increased relative abundance of *Sneathia* were risk factors for subsequent preterm pre-labor rupture of fetal membranes. Freitas and collaborators (Freitas et al., 2017) observed that a higher abundance of *Mollicutes*, higher richness, and higher diversity of the VM was more likely to appear in women with spontaneous preterm birth compared with those in women who delivered at term. Moreover, Eckert and colleagues (Eckert et al., 2003) observed that a reduced vaginal abundance of *Lactobacillus* and bacterial vaginosis (BV) were related to early pregnancy loss (<6 weeks) and spontaneous pregnancy (10–16 weeks) after IVF. However, the association between pregnancy location and microbial disorders is poorly understood.

Consistent with those findings, we revealed a positive relationship between a TP and lower abundance of *Lactobacillus* as well as an increased prevalence of bacteria of the genera *Gardnerella*, *Prevotella*, *Atopobium*, *Sneathia*, and *Megasphaera*. A causal relationship between VM alterations and pregnancy location is not clear, but two main mechanisms have been proposed (Shaw et al., 2011; Borges et al., 2014; Witkin and Linhares, 2017; Amabebe and Anumba, 2018; Greenbaum et al., 2019).

First, depletion of *Lactobacillus* weakens protection of the reproductive tract. *Lactobacillus* in the vagina is thought to have great significance for reproductive health. *Lactobacillus* may promote embryo implantation and pregnancy. *Lactobacillus* produce lactic acid and hydrogen peroxide. Lactic acid can acidify the vagina, which deters pathogen proliferation. In addition, lactic acid reduces the production of proinflammatory mediators to prevent pathogens causing infection and damage (Witkin and Linhares, 2017). As an oxidizing agent, hydrogen peroxide is toxic to anaerobes. *Lactobacillus* inhibits pathogen colonization by competitively occupying their potential binding sites in the vaginal epithelium (Borges et al., 2014; Greenbaum et al., 2019). *Lactobacillus* have been shown to prevent sexually transmitted infections (STIs) (Witkin and Linhares, 2017; Amabebe and Anumba, 2018). STIs, including those caused by *Neisseria gonorrhoeae*, *Chlamydia trachomatis*, and *Mycoplasma genitalium*, are most frequently linked with PID and TP, which can be explained by inflammation, fibrosis, and subsequent scarring of fallopian tube (Borges et al., 2014; Greenbaum et al., 2019). Besides, Shaw and colleagues (Shaw et al., 2011) found that *C. trachomatis* infection increased fallopian tube expression of PROKRS mRNA, resulting in ectopic implantation in the fallopian tube.

Second, bacteria of the genera *Gardnerella*, *Prevotella*, *Atopobium*, *Sneathia*, and *Megasphaera* are thought to be involved in BV pathogenesis (Coleman and Gaydos, 2018; Muzny et al., 2019). As the most common cause of vaginal discharge, BV is closely correlated with different adverse pregnancy outcomes (Cauci and Culhane, 2011; Kindinger et al., 2016). BV also acts as a risk factor of tubal-factor infertility (van Oostrum et al., 2013; Haahr et al., 2019), STIs (Wiesenfeld et al., 2003; Brotman et al., 2010; Molenaar et al., 2018), and PID (Haggerty et al., 2016; Ravel et al., 2021). Many BV-associated bacteria produce sialidases. Sialidases are thought to enhance ascending infection in the reproductive tract by hampering the host’s ability to recognize them, thereby facilitating bacterial attachment and inducing inflammatory reactions (Cauci and Culhane, 2011; Kindinger et al., 2016; Ravel et al., 2021).

Our study revealed the potential value of using the VM to classify the pregnancy location. However, translating these complex ecological metrics to the clinical setting is a challenge. Classification and categorization of the VM could greatly reduce the complexity of the biological dataset and be beneficial to epidemiological investigations and disease diagnoses (Cauci and Culhane, 2011; Kindinger et al., 2016; Ravel et al., 2021). Ravel and colleagues (Cauci and Culhane, 2011; Kindinger et al., 2016; Ravel et al., 2021) were the first to categorize the VM community in women of reproductive age based on the dominant bacterial species (using a threshold of 50% relative abundance**)** by clustering samples. That was a milestone study, and five types were found: four were dominated by *Lactobacillus* species (*L. crispatus, L. gasseri, L. iners*, and *L. jessenii*), and the remaining one was typified by a large proportion of anaerobic bacteria of genera *Prevotella, Dialister, Atopobium, Gardnerella, Megasphaera, Peptoniphilus, Sneathia, Eggerthella, Aerococcus, Finegoldia, and Mobiluncus*. Gajer and colleagues (Gajer et al., 2012) used hierarchical clustering to refine and improve this classification and proposed the term “community state types” (CSTs). Since then, CSTs have been applied widely in clinical research. Several studies have pointed out that women who have experienced preterm delivery would be classified as “*Lactobacillus*-poor CST 4” (DiGiulio et al., 2015; Tabatabaei et al., 2019; Chang et al., 2020).

However, CSTs have their limitations, one of which is that they are built on four definite dominant *Lactobacillus* species. Recent studies have found that, in early pregnancy, some VM communities are dominated by other or more than one type of *Lactobacillus* species. By contrast, classifications based on the relative abundance of *Lactobacillus* at the genus level could separate samples clearly and may appraise the relationship between the VM community and pregnancy outcomes more accurately (Haahr et al., 2019; Al-Memar et al., 2020; Chang et al., 2020). Besides, there is no exact method, standard clustering algorithm, or consentaneous threshold for CST assignment, and the different rules for CST generation result in different grouping outcomes (Koren et al., 2013; Loeper et al., 2018). Robinson and coworkers (Robinson et al., 2016) pointed out that using machine-learning algorithms instead of clustering for classification could overcome such drawbacks. Machine learning has improved bioinformatics analysis dramatically for making the microbial-community groups independent of samples and aiding comparability between studies (LeCun et al., 2015). Nevertheless, few studies have applied machine learning to VM classification.

Moreno and collaborators (Moreno et al., 2016) selected the relative abundance of four genera in the endometrial fluid as variables. Then, they applied two supervised machine-learning models—a CART model and a generalized linear model by logistic regression—to predict the pregnancy outcome of women undergoing IVF. Both models came to the same conclusion: *Lactobacillus* was the only available genus. Based on CART, the endometrial microbiota could be classified into two groups: LDM and NLDM. A receptive endometrium with NLDM in women undergoing IVF tended to produce poor pregnancy outcomes.

In this context, to establish an effective and robust classification, we undertook LEfSe to obtain potential vaginal taxonomic biomarkers. LEfSe is an algorithm which emphasizes statistical significance and biological relevance for identification of high-dimensional biomarkers (Segata et al., 2011). We found bacteria of the genus *Lactobacillus* to be significantly enriched in IUPs whereas bacteria of the genera *Gardnerella* and *Prevotella* were enriched in TPs. Then, we undertook five trials of 10-fold cross-validated CART to establish VM community groups. CART is a powerful machine-learning algorithm. On account of its validity for categorizing subjects in groups, as well as handling the multicollinearity and interactions of variables, CART has become increasingly popular in clinical research (**Marshall, 2001**; **Lemon et al., 2003**; **Henrard et al., 2015**). We showed that the relative abundance of *Lactobacillus* could be used to discriminate a TP from an IUP with high accuracy. Also, the VM could be classified into LDM and NLDM. Establishment of a classification system for the VM could simplify microbial structure.

The was the first comparative study of VM between IUP and TP groups using NGS. We also established a readily accessible model which could accurately classify a TP before a diagnosis using transvaginal ultrasound based on identification of key taxa and a machine-learning model. Furthermore, we used a specific population of clinical interest—a group of women with symptomatic early PUL—presenting for care. We used a well-defined study population, so the results will be limited to women with pain and/or uterine bleeding. Also, we assessed Chinese women; studies have pointed out that ethnicity can impact the VM composition (Ravel et al., 2011; MacIntyre et al., 2015; He et al., 2019; Serrano et al., 2019). Also, our study was associative; we focused on describing the different VM compositions between IUPs and TPs. Although we suggest that the relative abundance of *Lactobacillus* might be a valuable biomarker to classify a TP, the causality and mechanism remains to be determined. Longitudinal studies are required to demonstrate a causal relationship between the VM and pregnancy location. Hence, further large-scale studies that incorporate asymptomatic populations as well as different ethnic groups are needed. Moreover, due to the limitation of 16S rRNA gene sequencing, it cannot distinguish microbiota well at the species level and cannot identify other important organisms such as viruses and fungi.

## Conclusions

We showed, for the first time, that a higher evenness, greater diversity, and lower abundance of *Lactobacillus* in the VM was associated with a TP. The relative abundance of *Lactobacillus* in the VM could be a diagnostic marker. Our data provide a first glimpse and “snapshot” of the VM in early pregnancy, offering groundwork for further studies.

## Data Availability Statement

The data presented in the study are deposited in the Sequence Read Archive, accession number PRJNA737055.

## Ethics Statement

Our study was approved by the Ethical Committee of First Affiliated Hospital of Guangzhou University of Chinese Medicine (ZYYECK2017-060, approval date 06/02/2018), and all participants provided written informed consent. The patients/participants provided their written informed consent to participate in this study.

## Author Contributions

JG, G-PD, and S-PL designed and founded the study. Y-XH and X-JL recruited participants. X-FR, Y-XZ, SC, and X-RL collected clinical data and samples and analyzed and interpreted data. X-FR, Y-XZ, and SC generated figures and tables. X-FR wrote the first draft, which was developed by Y-XZ, F-FZ, and SC. All authors contributed to the article and approved the submitted version.

## Funding

Our study was supported by the National Natural Science Foundation of China (81774358 and 81373672), Post Project of Pearl River Scholar in Guangdong Province (A1-AFD018191Z0103), Traditional Chinese Medicine Bureau of Guangdong Province (Letter of Guangdong Traditional Chinese Medicine [2015] No. 19 and [2019] No. 5), and Guangzhou University of Chinese Medicine (XK2019016, 2019KYTD202 and 2019 II T33), Qi Huang Scholar in China (Letter of Chinese Traditional Medicine Education [2018] No.284), Research Projects in Key Fields of Guangdong Province (2020B1111100003).

## Conflict of Interest

The authors declare that the research was conducted in the absence of any commercial or financial relationships that could be construed as a potential conflict of interest.

## Supplementary Material

Supplementary Figure 1 Diversity measures in subjects with non-*Lactobacillus* dominated microbiota and with *Lactobacillus* dominated microbiota. **(A)** Alpha diversity represented by PD-whole-tree and Shannon diversity index in subjects with NLDM and LDM. Boxes with inside line represented interquartile range (IQR) and median, whiskers represented values within 1.5 × IQR of the first and third quartiles, points represented individual subjects. *P* was calculated by Wilcoxon test. **(B)** Principal coordinate analysis (PCoA) based on weighted UniFrac distances between the subjects with NLDM and LDM. Points represented individual subjects, and ellipses represented 95% confidence intervals around the cluster centroid. ANOSIM calculated *R* and *P* to determine the significance of clustering. Green indicated NLDM, orange indicated LD
