# Supplementary figures and images for: Non-Lactobacillus-Dominated Vaginal Microbiota Is Associated With a Tubal Pregnancy in Symptomatic Chinese Women in the Early Stage of Pregnancy: A Nested Case–Control Study
Source: Front Cell Infect Microbiol. 2021 Jul 7;11:659505. doi: 10.3389/fcimb.2021.659505 (PMC8294389; doi:10.3389/fcimb.2021.659505)

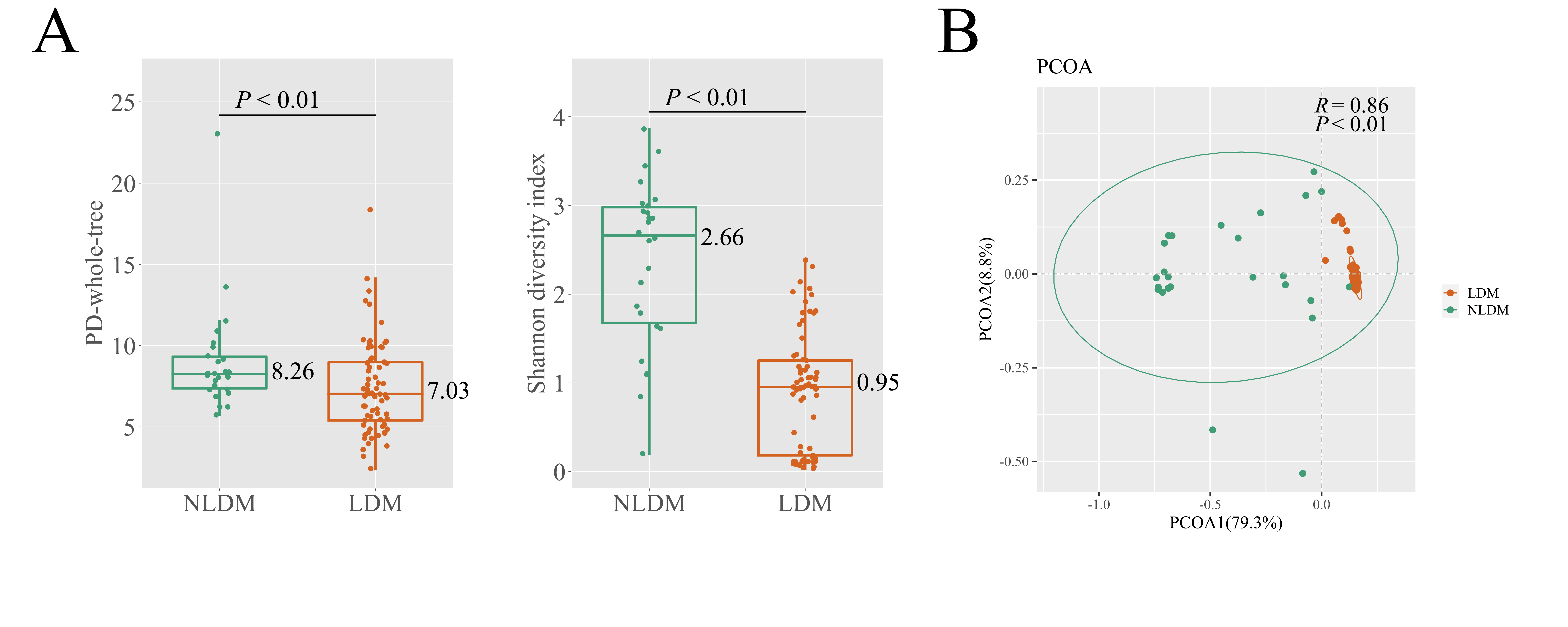

Supplement: Supplementary Figure 1 — Diversity measures in subjects with non-Lactobacillus dominated microbiota and with Lactobacillus dominated microbiota. (A) Alpha diversity represented by PD-whole-tree and Shannon diversity index in subjects with NLDM and LDM. Boxes with inside line represented interquartile range (IQR) and median, whiskers represented values within 1.5 × IQR of the first and third quartiles, points represented individual subjects. P was calculated by Wilcoxon test. (B) Principal coordinate analysis (PCoA) based on weighted UniFrac distances between the subjects with NLDM and LDM. Points represented individual subjects, and ellipses represented 95% confidence intervals around the cluster centroid. ANOSIM calculated R and P to determine the significance of clustering. Green indicated NLDM, orange indicated LD [file Image_1.jpeg]
